# Physical activity (20 min) is a powerful adjunct to insulin for correcting hyperglycaemia in Type 1 diabetes: A paradigm shift

**DOI:** 10.1111/dme.70163

**Published:** 2025-11-05

**Authors:** John S. Pemberton, Catherine L. Russon, Richard M. Pulsford, Brad S. Metcalf, Emma Cockcroft, Michael J. Allen, Anne M. Frohock, Rob C. Andrews

**Affiliations:** ^1^ Birmingham Women's and Children's Hospitals NHS Foundation Trust Diabetes Centre, Steelehouse Lane Birmingham UK; ^2^ University of Exeter Medical School Exeter UK; ^3^ Oxford University Hospitals NHS Foundation Trust Oxford UK; ^4^ NIHR Exeter Clinical Research Facility Exeter UK

Achieving target glucose remains one of the most persistent challenges in Type 1 diabetes (T1D),^1^ especially postprandially, where insulin cannot match rapid carbohydrate absorption.^2^ In our recent publication in *Diabetic Medicine*, we applied a causal matched‐pairs analysis to continuous glucose monitoring data, enabling comparisons of periods with and without physical activity under otherwise equivalent conditions.^3^ When glucose was above 10 mmol/L (180 mg/dL), about 20 minutes of everyday activity lowered levels by approximately 2 mmol/L (40 mg/dL), with hypoglycaemia risk under 2%. These findings support a simple heuristic for education—‘20 by 2’ in mmol/L, or ‘20 by 40’ in mg/dL—reframing physical activity as an acute, real‐time adjunct to insulin therapy for hyperglycaemia.

This commentary places these findings in historical and clinical context, highlights the methodological advance of causal inference through matched‐pair analysis and outlines the guardrails needed for safe translation into practice.

## 1 FROM RISK TO OPPORTUNITY

The glucose‐lowering effects of physical activity (PA) have been recognised for decades, but variability in individual responses has often been viewed as a barrier. In a pivotal analysis, Riddell and colleagues reported steep glucose declines in 120 adolescents with T1D undertaking 45–60 minutes of moderate walking or cycling within 4 h of prandial insulin, with hypoglycaemia in 44%.^4^ This gave rise to the familiar phrase, ‘the higher they start, the harder they fall’. Yet their secondary analysis, limited to PA events starting above 10.6 mmol/L (190 mg/dL) (*n* = 41), found that activity was effective at rapidly bringing glucose back into range, with hypoglycaemia risk under 10%.^3^

## 2 FROM EDUCATION TO CLINICAL TRANSLATION

The first structured approach using PA to reduce hyperglycaemia was introduced at Birmingham Children's Hospital in 2019. Building on Riddell's findings,^4^ young people and families were taught: if glucose is above 10 mmol/L, ‘15 minutes lowers it by 2 mmol/L’. In 2023, evaluation of this programme showed that those most engaged achieved the greatest time in range (TIR 3.9–10.0 mmol/L; 70–180 mg/dL) without more hypoglycaemia.^5^

## 3 SCALING UP THE EVIDENCE

The next step was to test whether these findings held in larger, more diverse populations. The Type 1 Diabetes Exercise Initiative (T1DEXI) adult and paediatric cohorts (T1DEXIP) provided this opportunity. Analysis of nearly 2000 bouts lasting 10–60 minutes confirmed that when started above 10 mmol/L, PA consistently lowered glucose into the target range.^6^ This effect was consistent across age, sex, regimen and activity type, establishing real‐world evidence for PA in correcting hyperglycaemia.^6^

But this analysis lacked a control condition. To address this, in our analysis in this edition of *Diabetic Medicine*, we applied causal modelling via a within‐subject matched‐pairs framework, focusing on PA bouts of 10–30 minutes when starting above 10 mmol/L.^3^ Each event was matched to a control period from the same individual, balanced on the four strongest predictors of glucose change: (i) starting glucose, (ii) glucose rate of change, (iii) insulin on board and (iv) preceding glucose variability. Robust matching across >1500 events, with balance confirmed by a standardised mean difference (SMD) of <0.001, established PA as the key determinant.

The analysis showed that 20 minutes of PA lowered glucose by approximately 2 mmol/L, an effect around 8‐fold greater than matched control periods. Hypoglycaemia during or immediately after PA was very rare (less than 2%).^3^ These findings provide the first causal‐style evidence that short bouts of everyday activity can be prescribed as an acute glucose‐lowering intervention for people with T1D from age 12, across insulin regimens and activity types.^3^ With evidence now secure, the next question is how to use PA safely in everyday practice.

## 4 SAFETY CONSIDERATIONS

As with any therapy, PA requires guardrails. Two prequalifying questions emerge from the evidence:

1. *If glucose >15.0 mmol/L (270 mg/dL), are ketone levels below thresholds of concern?* For pump users, the recommended threshold is <0.6 mmol/L (<+ on a urine strip); for multiple daily injections, <1.5 mmol/L (<++ on a urine strip). Consensus guidelines from the European Association for the Study of Diabetes (EASD) and the International Society for Pediatric and Adolescent Diabetes (ISPAD) advise against PA when glucose is >15 mmol/L (270 mg/dL) with elevated ketones and recommend insulin correction and extra monitoring.^7^
2. *Has insulin been administered in the past 4 h?* PA is most effective when performed within 4 h of a bolus insulin dose.^3^, ^4^, ^6^ ‘Insulin on board’ (IOB) is often used as a proxy for residual action, but calculators only imperfectly reflect pharmacokinetics.^8^ A more practical check is simply to ask whether insulin has been delivered in the preceding 4 h, reflecting the IOB linear model used in T1DEXI analyses^3^, ^6^ and the timeframes within which PA was undertaken in prior studies.^4^ If yes, PA functions as a potent adjunct to insulin.

## 5 POSITIONING ALONGSIDE OTHER ADJUNCT THERAPIES

PA should also be considered alongside other adjunct therapies. The newer glucagon‐like peptide‐1 receptor agonists (GLP‐1RAs) and dual agonists are emerging as leading candidates. For example, semaglutide (Ozempic®/Wegovy®) has been shown to improve glycaemic control, reduce weight and reduce insulin requirements when added to automated insulin delivery systems in adults with T1D.^9^ A recent consensus outlined how GLP‐1RAs could be integrated into care pathways.^10^ These developments indicate a shift towards multimodal care. Within this model, PA is distinctive—safe, cost‐free, accessible and deployable in real time with CGM.

## 6 CONCLUSION: A PRAGMATIC HEURISTIC

**FIGURE 1:**
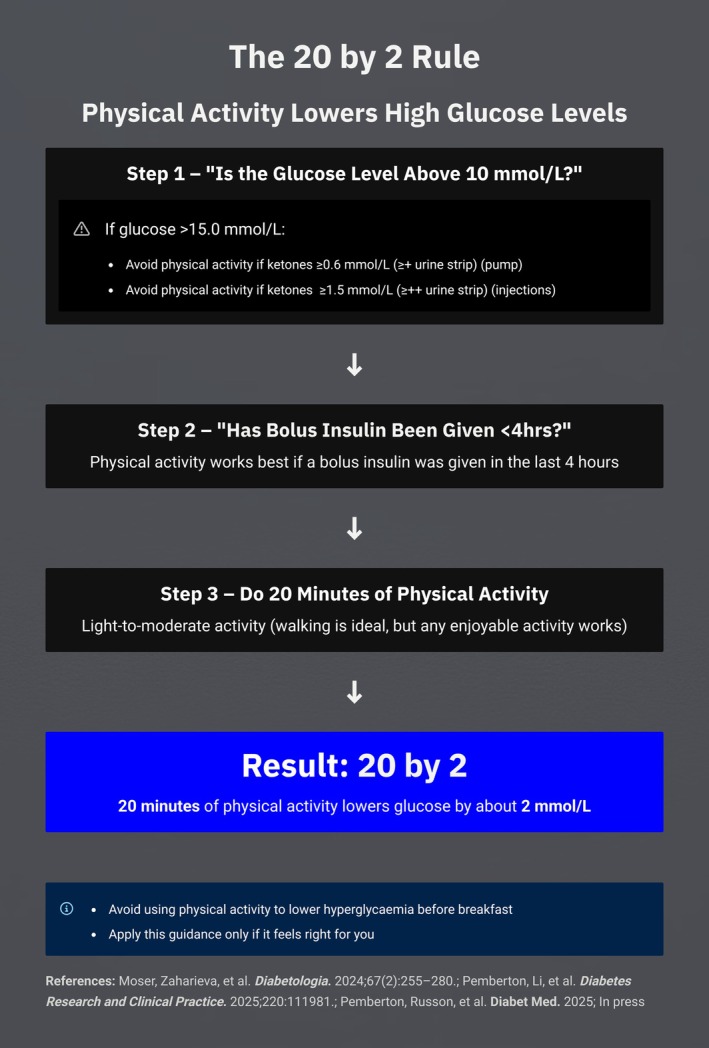
The 20 by 2 rule for lowering hyperglycaemia with physical activity (mmol/L format) International equivalent of the 20 by 40 rule, showing that 20 minutes of light‐to‐moderate activity can reduce glucose by approximately 2 mmol/L when initial levels exceed 10 mmol/L. Physical activity should not be used to correct hyperglycaemia before breakfast or when ketones are elevated.

**FIGURE 2:**
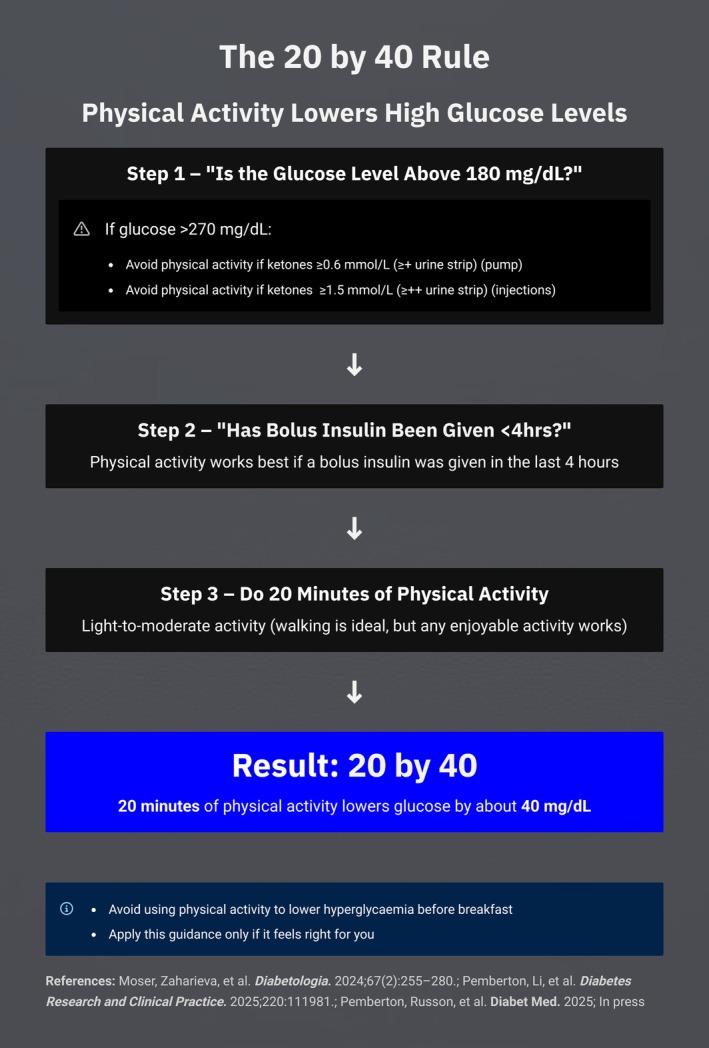
The 20 by 40 rule for lowering hyperglycaemia with physical activity. When glucose exceeds 180 mg/dL (10 mmol/L) and ketones are absent, 20 minutes of light‐to‐moderate activity such as walking typically lowers glucose by about 40 mg/dL. The effect is most pronounced if a bolus insulin dose was given within the previous 4 hours. Activity should be avoided if ketones are ≥0.6 mmol/L for pump users or ≥1.5 mmol/L for injection users.

The evidence supports a simple rule: when glucose is above 10 mmol/L, and provided that (i) bolus insulin has been delivered in the last 4 h, and (ii) if above 15.0 mmol/L (270 mg/dL), ketones are not elevated [≥ 0.6 mmol/L (≥ + on a urine strip) on pump therapy or >1.5 mmol/L (> ++ on a urine strip) otherwise], then 20 minutes of almost any activity will lower glucose by approximately 2 mmol/L (40 mg/dL). This ‘20 by 2’ (Figure 1) or ‘20 by 40’ mg/dL (Figure 2) principle is reproducible across cohorts, therapies and demographics. While longer durations of activity may further reduce glucose, they also increase the likelihood of hypoglycaemia, particularly when insulin on board is present. Therefore, longer durations should be accompanied by more vigilance.

Reframing physical activity as a powerful, real‐time glycaemic optimiser—rather than only a long‐term health strategy—positions it as a safe, zero‐cost therapy that, in the era of continuous glucose monitoring, delivers instant feedback and reinforces a virtuous cycle of activity driving better control. Future research should also explore whether short bouts of activity can be used pre‐emptively to prevent post‐prandial glucose excursions, in addition to their corrective role when glucose is elevated.

## AUTHOR CONTRIBUTIONS

John Pemberton: Conceptualisation, Background research, Writing – original draft. Catherine L. Russon: Writing – review and editing. Richard Pulsford: Writing – review and editing. Bradley S. Metcalf: Writing – review and editing. Emma Cockroft: Writing – review and editing. Michael Allen: Analysis, Writing – review and editing. Anne‐Marie Frohock: Writing – review and editing. Robert C. Andrews: Supervision, writing – review and editing, intellectual revision.

## CONFLICT OF INTEREST STATEMENT

John Pemberton reports being on the advisory board for Abbott and ROCHE and speaker fees from Abbott, Dexcom and Insulet in the last 3 years. Faculty member of Exercise for Type 1 Diabetes. Catherine L. Russon, Richard Pulsford, Bradley S. Metcalf, Emma Cockroft, and Michael Allen have no conflicts. Anne‐Marie Frohock reports consultancy fees for Insulet and speaker fees from Dexcom and Insulet in the last 3 years. Faculty member of Exercise for Type 1 Diabetes. Robert C. Andrews reports research funding from NovoNordisk Healthcare Organisation in the last 3 years, honoraria from NovoNordisk, AstraZeneca and Eli Lilly for education talks on diet and exercise to health care professionals. Co‐founder of Exercise for Type 1 Diabetes.

## GUARANTOR STATEMENT

John Pemberton is the guarantor of this work and, as such, had full access to all the data in the study and takes responsibility for the integrity of the data and the accuracy of the data analysis.

## PRIOR PRESENTATION

Accepted for oral presentation at the European Association for the Study of Diabetes (EASD) Annual Meeting 2025, Vienna, Austria.
